# Incidence, risk factors, and clinical findings of syphilis among men living with HIV in Croatia during the COVID-19 pandemic

**DOI:** 10.1038/s41598-023-38807-1

**Published:** 2023-07-21

**Authors:** Josip Begovac, Vanja Romih Pintar, Nina Vrsaljko, Loris Močibob, Nikolina Bogdanić, Šime Zekan, Oktavija Đaković Rode

**Affiliations:** 1 University Hospital for Infectious Diseases “Dr. Fran Mihaljević”, Zagreb, Croatia; 2 School of Medicine, University of Zagreb, Zagreb, Croatia; 3 School of Dental Medicine, University of Zagreb, Zagreb, Croatia

**Keywords:** Bacterial infection, HIV infections

## Abstract

We conducted a nationwide longitudinal observational study to estimate the incidence of syphilis in a cohort of male persons living with HIV (MLWH) in Croatia in the pre-COVID-19 and COVID-19 years. Data were reviewed and extracted from the clinical database. We analyzed 1187 MLWH (≥ 18 years) in care in Croatia from 2018 to 2021 and used Poisson regression to calculate rates. We observed a 91.4% increase in incidence between 2019 and 2020; the overall rate was 6.0/100 person-years, and the annual rate ranged from 3.3/100 person-years in 2018 to 9.3/100 person-years in 2021. We found higher rates in men who have sex with men, MLWH with a baseline history of syphilis, MLWH with a more recent HIV diagnosis, and a lower rate in those who had clinical AIDS. The rate of syphilis serological testing was 3.5% lower in 2020 compared to 2019. Recurrent syphilis was more likely asymptomatic compared to the first episodes. In conclusion, during the COVID-19 epidemic years, there was a huge increase in syphilis. Results highlight the need for enhanced and novel prevention interventions.

## Introduction

Soon after the first reported case of the coronavirus disease (COVID-19) caused by a new strain of severe acute respiratory syndrome coronavirus 2 (SARS-CoV-2) in 2019 in China, the world faced a global health crisis. Considering all preventive measures to control the spread of SARS-CoV-2 infection through quarantine and social distancing, it was to be expected that there would be changes in sexual behaviors leading to a decrease in the incidence of sexually transmitted diseases (STDs). However, the data on STDs during the COVID-19 epidemic years is still evolving and the impact of the COVID-19 pandemic on STDs remains to be defined and might be different in different countries. Some studies reported a decrease in the number of reported STDs particularly during the lockdown periods^1–6^, whereas other studies found no significant change^7,8^ or an increase in the number of reported STDs, including syphilis^9–16^.

*Syphilis* is a highly contagious bacterial infection caused by gram-negative spirochaete *Treponema pallidum* and is typically spread through sexual contact. The World Health Organization estimated that seven million people got infected with syphilis worldwide in 2020^17^, the highest incidence was recorded among men who have sex with men (MSM)^18^.

In the most recent European Centre for Disease Prevention and Control (ECDC) surveillance report 35,039 confirmed syphilis cases were reported in 29 EU/EEA Member States, with a crude notification rate of 7.4 cases per 100,000 population^19^. The majority of cases (73%) were reported in MSM, and the trends in MSM showed a steep increase in reported cases from 2010 up to 2017, with a much slower increase between 2018 and 2019^19^. Syphilis is a notifiable disease in Croatia, however, because of underreporting of cases the notification reports from the Croatian Nation Public Health Institute do not seem reliable. In the ECDC report the annual number of reported cases of syphilis ranged from 25 in 2015 to 35 in 2018 in Croatia. There have been two studies that estimated the prevalence of past syphilis among MSM measured by *Treponema pallidum* hemagglutination in Croatia. Both studies used a respondent-driven sampling method and reported a prevalence of syphilis of 10.6% in 2006 and 7.6% in 2011^20^. The aim of this study was to estimate the incidence of syphilis diagnoses in a cohort of male persons living with HIV (MLWH) in Croatia in the pre-COVID-19 and COVID-19 years and to identify syphilis risk factors in this population.

## Methods

### Setting

In Croatia, all people living with HIV (PLWH) are treated at the University Hospital for Infectious Diseases (UHID) in Zagreb. The HIV Outpatient Department at UHID has been established in 1997, it currently provides outpatient care for PLWH including treatment for common STDs such as gonorrhea, chlamydia, and syphilis. Antiretroviral therapy is also given from the hospital pharmacy. Because of the distance from the residence of PLWH to the treatment center, phone consultations were part of routine care since 1997 and a mHealth solution delivering laboratory results to the smartphones of PLWH has been introduced in 2016^21^. Antiretroviral therapy (ART) can also be delivered to the home address, this is usually done after a phone consultation.

Serologic testing for syphilis at UHID is usually routinely done annually or bi-annually, however, it may be done more or less frequently depending on the clinical findings and sexual behavior history. Since 2004, the testing algorithm for syphilis screening includes first a treponemal test (the *Treponema pallidum* hemagglutination assay [TPHA]), followed by a nontreponemal test (the Rapid Plasma Regain [RPR] test).

### Population and definitions

**Figure 1:**
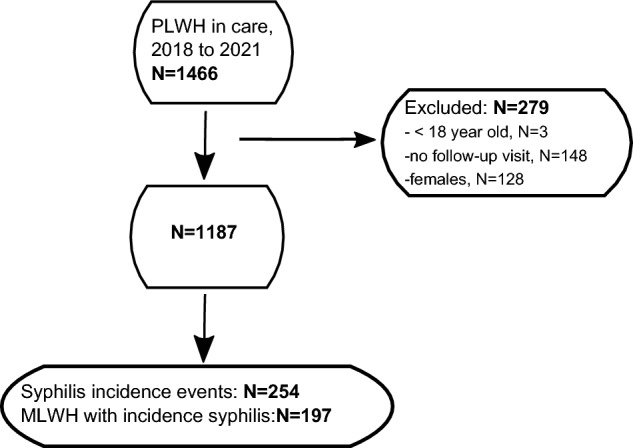
Flow diagram of participants included into the study. PLWH, people living with HIV; MLWH, men living with HIV.

Because no women had syphilis, we restricted our analysis to men (Fig. 1) and since there were only 3 transgender women, we refer to men as males at birth. Included were men (> 18 years) living with HIV receiving care at UHID with at least 1 year of follow-up (n = 1187, Fig. 1). The observation period started on or after January 1st, 2018, and ended on December 31st, 2021, or earlier if MLWH died, moved, or were lost to follow-up. The person’s data, including demographic characteristics, diagnoses, prescribed medications, number of syphilis tests, and laboratory values, were reviewed and extracted from the electronic database. Diagnoses of primary, secondary, early latent (infection within 1 year) and late latent syphilis (infection > 1 year), and neurosyphilis were made by clinicians at UHID. A syphilis clinical diagnosis typically included a history of exposure, physical findings (e.g. ulcers, rash, lymphadenopathy, oral mucosal erosions, etc.), and serological tests.

Incident syphilis was defined as a new syphilis diagnosis, it included both first-time and reinfection events. We defined incident cases of syphilis based on clinical diagnosis, prescribed treatment, and serological testing. Syphilis was considered present if any of the following were observed: both a documented syphilis clinical diagnosis and treatment for syphilis, a positive syphilis TPHA screen when a previous test was negative, and a four-fold increase in RPR titer. Episodes of syphilis at inclusion into the study (n = 18) were not considered incident cases. Past history of syphilis at inclusion into care was defined if treatment was recalled by the patient or the TPHA test was positive.

The Ethics Committee of the University Hospital for Infectious Diseases (UHID), Zagreb, Croatia approved the study (No. 01-1931-7-2020). The study was performed following the Declaration of Helsinki and the Code of Medical Ethics and Deontology of the Croatian Medical Chamber. Written informed consent was obtained from all participants included in this study.

### Variables

We examined age, HIV transmission risk (MSM, heterosexual, injection drug use, other/unknown), calendar year of diagnosis, clinical AIDS, living in Zagreb or Zagreb County, baseline and follow-up HIV-1 RNA, baseline CD4 cell count, baseline antiretroviral therapy and hepatitis C and B infection. Of note, the MSM risk categories included 3 transgender women who have had sex with men. The major outcome of interest was a new syphilis diagnosis.

### Statistical analysis

We used descriptive statistics to characterize our study population. Baseline socio-demographic, laboratory, and clinical characteristics were compared between MLWH with and without incident syphilis with chi-square tests or Fisher’s exact tests for categorical variables and Wilcoxon rank-sum tests for continuous variables. The chi-square trend test was used to examine the proportion of asymptomatic syphilis cases from 2018 to 2021. We also examined whether the first syphilis episode after inclusion into care was more likely asymptomatic in MLWH with a baseline history of past or current syphilis.

Using Poisson regression, we calculated crude incidence rates for incident syphilis (first and repeated episodes) by sociodemographic and clinical characteristics. To examine factors related to syphilis diagnoses, incident rate ratios with 95% confidence intervals (CI) were calculated. The calendar year was annualized, and we used a generalized estimating equations (GEE) model with an exchangeable correlation structure to account for recurrent events. Age, viral load, prior episode of syphilis, clinical AIDS, and hepatitis C infection on follow-up were time-updated variables whereas other variables were fixed. We modeled the total number of events using an offset for the person-year follow-up time. Crude analyses included the outcome and one predictor variable whereas multivariable analyses included more than one predictor. Statistical analyses were done using SAS version 9.4 (SAS Institute, Inc., Cary, North Carolina). P-values were two-sided and statistical significance was set at the conventional P-value of < 0.05.

### Ethical approval

The study was approved by the University Hospital of Infectious Diseases Ethics Committee.

## Results

### Descriptive analysis

Of 1187 males at birth, three were transgender women and all, except one man, were Caucasians. The HIV transmission risk was: sex between men (n = 976, 82.2%), heterosexual (n = 142, 12.0%), injection drug use (n = 28, 2.4%), recipient of blood or blood products (n = 5, 0.4%), mother to child transmission (n = 4, 0.3%) and unknown (n = 32, 2.7%). At baseline, the median age was lower among MLWH who had incident syphilis compared to those who did not (40.4 years *versus* 42.0 years) (Table 1). Median age and different age categories at a syphilis diagnosis on follow-up are presented in the Supplementary Material. MLWH who had syphilis on follow-up were more likely MSM, living in Zagreb, diagnosed with HIV in the period 2016 to 2020, and did not have clinical AIDS (Table 1). The median CD4 lymphocyte count at baseline was high (599.5 per mm^3^), 75.2% had < 50 copies of HIV1-RNA per ml, and 91.8% were ART-experienced (Table 1). Of 284 MLWH who had clinical AIDS, 278 had clinical AIDS in the baseline calendar year and 6 had clinical AIDS in a follow-up calendar year. A total of 67 MLWH had hepatitis C infection (HCV), 62 at baseline, and 5 acquired HCV infection during follow-up. There were no new hepatitis B infections during the follow-up.

**Table 1:** Main baseline characteristics of 1187 men living with HIV with and without incident syphilis on follow-up, Croatia, 2018–2021.

	Incident syphilis			
	No (N = 990)	Yes (N = 197)	Total (N = 1187)	*P* Value
Age, years	42.0 (35.0–51.2)	40.4 (32.4–46.7)	41.7 (34.6–50.4)	0.001
Age groups, years				0.014
18–29	136 (13.7)	26 (13.2)	162 (13.6)	
30–39	280 (28.3)	70 (35.5)	350 (29.5)	
40–49	301 (30.4)	66 (33.5)	367 (30.9)	
50–59	171 (17.3)	28 (14.2)	199 (16.8)	
60+	102 (10.3)	7 (3.6)	109 (9.2)	
Mode of transmission				< 0.001
MSM	789 (79.7)	187 (94.9)	976 (82.2)	
Heterosexual	137 (13.8)	5 (2.5)	142 (12.0)	
Other/unknown	064 (6.5)	5 (2.5)	69 (5.8)	
Year of HIV diagnosis				< 0.001
≤ 2010	405 (40.9)	48 (24.4)	453 (38.2)	
2011–2015	282 (28.5)	62 (31.5)	344 (29.0)	
2016–2020	303 (30.6)	87 (44.2)	390 (32.9)	
Known duration of HIV infection, years	5.0 (1.2–10.8)	3.2 (0.4–6.7)	4.6 (1.1–10.3)	< 0.001
Living in Zagreb				< 0.001
No	540 (54.5)	78 (39.6)	618 (52.1)	
Yes	450 (45.5)	119 (60.4)	569 (47.9)	
Had clinical AIDS^a^				< 0.001
No	736 (73.8)	173 (87.8)	909 (76.6)	
Yes	254 (26.2)	24 (12.2)	278 (23.4)	
Receiving antiretroviral therapy				0.77
Before baseline	911 (92.0)	179 (90.9)	1090 (91.8)	
After baseline	72 (7.3)	17 (8.6)	89 (7.5)	
No ART	7 (0.7)	1 (0.5)	8 (0.7)	
Baseline HIV1-RNA				0.10
≥ 50 c/ml	233 (23.8)	58 (29.4)	291 (24.8)	
< 50 c/ml	745 (76.2)	139 (70.6)	884 (75.2)	
Baseline CD4 cell count per mm^3^	598.0 (366.5–811.5)	620.5 (425.5–810.5)	599.5 (374.0–810.5)	0.25
Baseline CD4 cell categories count per mm^3^				0.23
> 500	576 (61.3)	129 (65.8)	705 (62.1)	
≤ 500	364 (38.7)	67 (34.2)	431 (37.9)	
Antibody to hepatitis C				0.25
Negative	932 (94.4)	190 (96.4)	1125 (94.8)	
Positive	55 (5.6)	7 (3.6)	62 (5.2)	
Hepatitis B^b^				0.63
Negative	935 (94.7)	185 (93.9)	1122 (94.6)	
Positive	52 (5.3)	12 (6.1)	64 (5.4)	
Had syphilis at or before inclusion^c^				< 0.001
Yes	286 (28.9)	81 (41.1)	367 (30.9)	
No	704 (71.1)	116 (58.9)	820 (69.1)	

Men are males at birth, there were 3 transgender women. Values are frequencies with percentages and median with first and third quartile in parenthesis. MSM, men who have sex with men. ART, antiretroviral therapy.

^a^Clinical AIDS in the baseline calendar year.

^b^61 had chronic, 3 had acute hepatitis B.

^c^Based on history and/or positive *Treponema pallidum* hemagglutination assay.

### Longitudinal analysis

**Figure 2:**
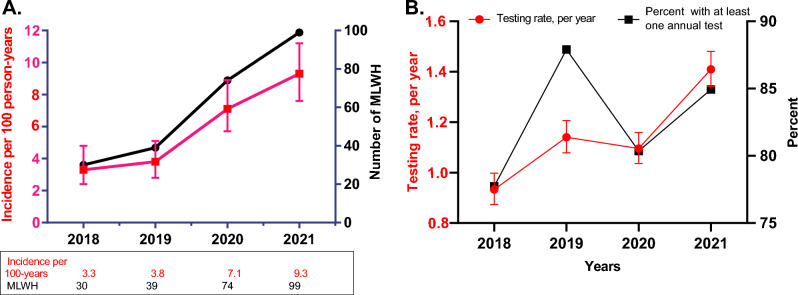
The increasing incidence of syphilis, per 100 person-years with 95% confidence intervals and number of MLWH with syphilis (**A**). Yearly rates with 95% confidence intervals of episodes of syphilis testing (left axis) and percent of men living with HIV who had at least one syphilis test in a calendar year (right axis) (**B**), Croatia, 2018–2021. MLWH, men living with HIV.

Of 1187 MLWH, 197 (16.6%) experienced 254 first-time or reinfection syphilis events during 4224.2 person-years of follow-up (PYFU). The median follow-up was 4.0 years, (first quartile [Q1] to third quartile [Q3], 3.3–4.0 years), and the overall incidence rate was 6.0/100 PYFU (95% confidence interval [CI] 5.3–6.8). Of 254 syphilis events, 116 (45.7%) were the first-ever diagnoses. The incidence rate for first-ever syphilis events was 5.0 (95% CI, 4.2–5.9) per 100 PYFU, and for the recurrent event, it was 8.3 (95%, 6.8–10.0) per 100 PYFU. There was an increase in the incidence of syphilis in the period 2018–2021 (Table 2, Fig. 2A). This increase was particularly high between 2019 and 2020 (91.4%, Table 3).

**Table 2:** Rates of incident syphilis in men living with HIV by sociodemographic and clinical characteristics and univariable and multivariable syphilis diagnosis, Croatia, 2018 to 2021.

Characteristics	Syphilis diagnoses	Person-years	Syphilis diagnoses per 100 person-years (95% CI)	Crude IRR (95% CI)	P value	Adjusted IRR (95% CI)	P value
Sociodemographic characteristics							
Age^a^, years							
18–39	112	1379.9	8.1 (6.7–9.8)	1.56 (1.18–2.06)	0.002	1.20 (0.89–1.61)	0.230
40+	142	2844.3	5.0 (4.2–5.9)	Referent			
HIV transmission risk							
MSM	243	3473.3	7.0 (6.2–7.9)	4.65 (2.45–8.82)	< 0.001	3.06 (1.53–6.11)	0.002
Other/unknown	11	750.9	1.5 (0.8–2.6)	Referent			
Living in Zagreb							
Yes	149	2024.9	7.4 (6.3–8.6)	1.65 (1.23–2.21)	0.001	1.29 (0.96–1.74)	0.088
No	105	2199.2	4.8 (3.9–5.8)	Referent			
Calendar year of follow up							
2021	103	1113.0	9.3 (7.6–11.2)	2.76 (1.84–4.15)	< 0.001	2.69 (1.78–4.05)	< 0.001
2020	80	1119.9	7.1 (5.7–8.9)	2.11 (1.38–3.23)	0.001	2.06 (1.35–3.15)	0.001
2019	40	1064.3	3.8 (2.8–5.1)	1.10 (0.72–1.70)	0.653	1.10 (0.71–1.70)	0.665
2018	31	927.0	3.3 (2.4–4.8)	Referent		Referent	
HIV characteristics							
Year of HIV diagnosis							
2016–2020	112	1700.2	9.1 (7.6–10.9)	2.45 (1.72–3.50)	< 0.001	1.58 (1.08–2.32)	0.018
2011–2015	83	1292.7	6.4 (5.2–8.0)	1.86 (1.27–2.73)	0.002	1.31 (0.88–1.94)	0.181
< 2010	59	1231.2	3.5 (2.7–4.5)	Referent		Referent	
Had clinical AIDS^a^							
Yes	31	1022.5	3.0 (2.1–4.3)	0.45 (0.28–0.72)	0.001	0.56 (0.35–0.89)	0.014
No	223	3201.7	7.0 (6.1–7.9)	Referent		Referent	
HIV–1 RNA < 50 c/ml^a^							
Yes	229	3544.3	6.5 (5.7–7.4)	1.17 (0.63–2.16)	0.623	b	
No	22	309.8	7.1 (4.7–10.8)	Referent			
Coinfections							
HCV antibody^a^						b	
Positive	9	244.5	3.7 (1.9–7.1)	0.58 (0.29–1.17)	0.130		
Negative	245	3979.7	6.2 (5.4–7.0)	Referent			
Chronic or acute hepatitis B^c^						b	
Yes	15	222.8	6.7 (4.1–11.2)	1.20 (0.68–2.12)	0.524		
No	239	4001.4	6.0 (5.3–6.8)	Referent			
Previous episode of syphilis^a,d^							
Yes	109	1320.5	8.3 (6.8–10.0)	1.68 (1.27–2.20)	0.001	1.41 (1.06–1.86)	0.018
No	145	2903.7	5.0 (4.2–5.9)				

Men were males at birth, there were 3 transgender women. MSM, men who have sex with men; IRR, incidence rate ratio; CI, confidence intervals; ^a^Time updated; ^b^Not included in the multivariable regression model. ^c^At baseline, ^d^Evidence of syphilis at baseline or on follow-up. IRR are based on a generalized estimating equations model with an exchangeable correlation structure. The overall incidence rate per 100 person-years was 6.0 (95% CI, 5.3–6.8). The 95% confidence intervals for the rate per 100 person-years of observation was calculated using the Rothman/Greenland estimation.

**Table 3:** Rates of new symptomatic and asymptomatic syphilis among men living with HIV, Croatia, 2018–2021.

Year	Symptomatic syphilis		Asymptomatic syphilis		Total syphilis events	
	Rate per 100 PY (95% CI)	Percent change from preceding year	Rate per 100 PY (95% CI)	Percent change from preceding year	Rate of syphilis per 100 PY (95% CI)	Percent change from preceding year
2018	2.2 (1.4–3.3)	–	1.2 (0.7–2.1)	–	3.3 (2.4–4.8)	–
2019	2.3 (1.5–3.4)	3.8	1.5 (0.9–2.5)	23.1	3.8 (2.8–5.1)	10.5
2020	4.6 (3.5–6.0)	106.1	2.6 (1.8–3.7)	70.2	7.1 (5.7–8.9)	91.4
2021	5.3 (4.1–6.8)	16.7	4.0 (2.9–5.3)	54.4	9.3 (7.6–11.2)	30.7

Men were males at birth, there were 3 transgender women.

Percent change from preceding year based on the incidence rate ratio from generalized estimating equations models. PY, person-years. CI, confidence interval.

Overall, the syphilis testing rate was 1.15 (95% CI, 1.12–1.19) per one person-year, in MSM the rate was 1.23 (95% CI, 1.19–1.26). The testing rate was lowest in 2018 (0.93, 95% CI, 0.87–1.00) and highest in 2021 (1.41, 95% CI, 1.34–1.48), however, the testing rate was similar in 2019 (1.14, 95% CI, 1.08–1.21) compared to 2020 (1.10, 95% CI, 1.04–1.16, *P* = 0.105 form the GEE model with Tukey–Kramer adjustment) (Fig. 2B). The proportion of MLWH who had at least one syphilis test per calendar year ranged from 77.7 to 87.9% and was 8.5% lower in 2020 compared to 2019 (Fig. 2B). The frequency of testing in the whole population and MSM is presented in more detail in Supplementary Tables 1S and 2S. Of 1187 men, 513 (43.2%) missed an annual syphilis test (Supplementary Table 3S). Those who had missed at least one annual syphilis test compared to those who had not were: older, acquired HIV more frequently by heterosexual transmission, had a longer history of ART, and more frequently lived outside Zagreb. Participants who had at least one annual syphilis test had more frequently past syphilis or syphilis at inclusion into the study (53 of 674) compared to those who did not have regular annual tests (28 of 513), but this difference was not statistically significant (P = 0.100, Supplementary Table 3S).

The number of syphilis events, follow-up time, events per 100 PYFU, and incidence rate ratios are shown in Table 2 and Supplementary Fig. S1. On crude analysis we observed evidence of a higher rate of a new episode of syphilis among MSM, those aged less than 40, those diagnosed with HIV and syphilis more recently, living in Zagreb and in MLWH who had past evidence of syphilis (Table 2, Supplementary Fig. S1). Syphilis diagnoses per 100 person-years were similar in the age time-updated groups 18–29 years (N = 26, rate = 6.9 [95% CI, 4.7–10.1]) compared to the 30–49 years (N = 169, rate, 7.0 [95% CI, 6.0–8.1]) and higher compared to those older than 50 (N = 59, rate, 4.2 [95% CI, 3.2–5.4]). On multivariable analysis, many of the relationships found on crude analysis were less pronounced, statistical significance was found for MSM *versus* others, more recent calendar year of HIV diagnosis (2016–2020 versus ≤ 2010), more recent calendar years of syphilis diagnosis, and having a previous episode of syphilis (Table 2, Supplementary Fig. S1). A previous clinical AIDS diagnosis was associated with a decreased risk of having syphilis on follow-up (Table 2, Supplementary Fig. S1). The annual rate of asymptomatic and symptomatic syphilis diagnoses and the percent increase compared to the previous years are shown in Table 3.

We performed several other analyses which included only observations with at least one syphilis test done in a calendar year or included only MSM (Supplementary Tables 4S–6S and Supplementary Figs. S2–S4). In those analyses, the rates of syphilis events were higher than in our main analysis. The overall rate per 100 PYFU was as follows: 7.2 (95% CI, 6.3–8.1) in all men who had a syphilis test done at least once annually, 7.0 (95% CI, 6.2–8.0) in MSM, and 8.1 (95% CI, 7.1–9.2) in MSM with annual syphilis tests done.

### Clinical presentation

**Figure 3:**
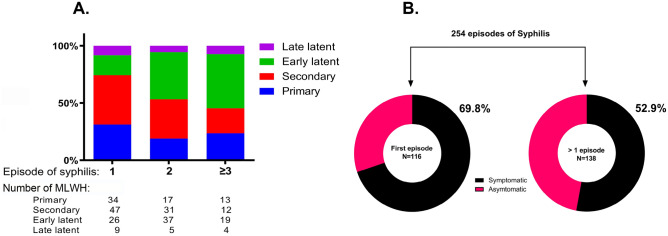
Syphilis stage during follow-up among individuals with 1, 2 or ≥ 3 total episodes of syphilis (**A**) and comparison of symptomatic and asymptomatic syphilis based on the first ever or repeated syphilis episode (**B**). Number of persons in each of the three stacked bars by syphilis stage are below panel (**A**). The proportion of persons with asymptomatic syphilis was higher in those with a repeated syphilis episodes compared to those with the first one (P = 0.006) (**B**). MLWH, men living with HIV.

The major clinical findings are described in Table 4. Overall, of 254 syphilis episodes 154 (60.6%) were symptomatic, secondary syphilis was diagnosed in 90 (35.4%), and primary in 64 (25.2%) cases (Table 4). The proportion of asymptomatic cases ranged from 35.5% in 2018 to 42.7% in 2021, however, this trend was not statistically significant (*P* = 0.483, chi-square trend test). The use of intramuscular benzathine penicillin G 2.4 million units in a single dose for the treatment of primary, secondary, and early latent syphilis increased from 2018 to 2021 (Table 4). Of 116 first syphilis episodes 81 (69.8%) were symptomatic whereas of 138 recurrent episodes 73 (52.9%) were symptomatic (*P* = 0.006) (Fig. 3A and B). MLWH with syphilis at baseline or a history of previous syphilis were more likely to have an asymptomatic first syphilis event on follow-up (OR 2.49, 95% CI, 1.38–4.49). The median number of serological tests before the first syphilis event on follow-up was the same in MLWH with a baseline history of syphilis and those who did not have past syphilis (3, Q1–Q3, 2–4).

**Table 4:** Major clinical findings and treatment of 254 episodes of syphilis in men, Croatia, 2018–2021.

Feature	Year			
	2018 (n = 31)	2019 (n = 40)	2020 (n = 80)	2021 (n = 103)
Symptomatic, yes	20 (64.5)	24 (60.0)	51 (63.8)	59 (57.3)
Stage				
Primary	11 (35.5)	9 (22.5)	20 (25.0)	24 (23.3)
Secondary	9 (29.0)	15 (37.5)	31 (38.8)	35 (34.0)
Early latent	7 (22.6)	12 (30.0)	27 (33.8)	36 (35.0)
Late latent	4 (12.9)	4 (10.0)	2 (2.5)	8 (7.8)
First episode	18 (58.1)	15 (37.5)	36 (45.0)	47 (45.6)
Rash	8 (25.8)	14 (35.00)	30 (37.5)	30 (29.1)
Condylomata lata	1 (3.2)	0 (0.0)	3 (3.8)	0 (0.0)
Clinical neurosyphilis	0	1 (2.5)	0	1 (1.0)
Treatment				
Penicillin, total	28 (90.3)	34 (85.0)	68 (85.00)	95 (92.2)
Penicillin, one shot^a^	19 (61.3)	26 (65.0)	64 (80.0)	85 (82.5)
Doxycycline	3 (9.7)	6 (15.0)	12 (15.0)	8 (7.8)

Men were males at birth, there were 3 transgender women. Values are frequencies and percentages in parentheses.

^a^Treatment of primary, secondary and early latent syphilis with one shot of benzathine benzylpenicillin 2.4 million I.U. intramuscular.

## Discussion

In this nationwide study of men living with HIV, the incidence of syphilis increased by 91.4% in 2020 compared to 2019, and this increase was mainly driven by an increase in symptomatic cases (106.1%) (Table 3). We also found a 30.7% increase in the incidence of syphilis in 2021 compared to the previous year, but this increase was larger in asymptomatic syphilis cases. In our models, the incident rate of syphilis was at least > 2.5 times higher in 2021 compared to 2018. The increase in syphilis between 2020 and 2019 occurred in the background of a somewhat higher syphilis testing rate in 2019 (1.14 tests per one person-years) compared to 2020 (1.10 tests per one person-years).

The overall incidence rate of syphilis in our men living with HIV was 6.0/100 PYFU and the annual incidence rate ranged from 3.3/100 PYFU in 2018 to 9.3/100 PYFU in 2021. Similarly, to our findings in 2021, a retrospective cohort study from Australia reported an incidence of early syphilis of 9.3 per 100 person-years in MSM living with HIV in the period 2013–2019^22^. Another study from Thailand found an incidence of syphilis of 10.2 per 100 person-years in a predominantly MSM cohort diagnosed with acute HIV infection in the period April 2009 through December 2018^23^. A very high incidence of syphilis in PLWH was reported from Mexico (16.0/100 PYFU, years covered: 2011–2015)^24^ and in MSM living with HIV from Argentina (14.9/100 PYFU) in the period 1 March 2015–29 February 2016^25^. Some studies reported somewhat lower incidence rates in PLWH than ours. A study from the HIV Swiss cohort reported data from October 2017 to November 2019 and found an incidence of syphilis of 4.4 cases per 100 person-years in male PLWH and of 6.3 cases per 100 person-years in MSM^26^. A recent analysis from the French Dat’AIDS HIV cohort including men and women, found an incidence of syphilis of 3.8 per 100 person-years for infection and 6.5 per 100 person-years for re-infection in the period 2010–2019^27^. Data from four US clinical sites reported an incidence rate of 5.6/100 PYFU in men and 6.9/100 PYFU in MSM in the period 2014–2018^28^. There are reports on an increasing number of cases of syphilis in some countries during the COVID-19 years 2020 and 2021^9–11^, however, data on the incidence density rates are lacking.

This huge increase in syphilis in MLWH occurred in Croatia one to two decades after similar outbreaks were reported in North America and Western Europe. Outbreaks of syphilis among MSM have been reported from the US since 2000^29^ and ECDC reported a sharp increase (> 50%) of notifiable cases in several European countries (Germany, Denmark, Greece, Luxembourg, Malta, Norway, Portugal, and Sweden) between 2008 and 2013^30^. This increase in syphilis cases in Europe continued for at least up to 2019^19^. ECDC reported that 68% of syphilis cases were in MSM in 2019 and that among the countries that reported the HIV status 43% of cases were in PLWH^19^. It has been suggested that the high incidence of syphilis among MSM and PLWH could be due to risk perception, risky sexual behaviors such as unprotected anal sex, occasional sexual partners, concurrent sexual partners, HIV serosorting, chemex, transactional sex, methamphetamine use, group sex or contacting partners over the internet, and anonymous couples^31–35^. Closed sexual networks are important contributors to syphilis outbreaks^36,37^. As we have not collected data on sexual risks, we do not know whether those practices changed during the COVID-19 pandemic. However, lockdown measures and measures of social distancing with other precautions might have given a false sense of security. In Croatia, the sexual network dynamic may have changed during 2019 and 2020 leading to a local syphilis epidemic. There is little data on risk behaviors in PLWH from Croatia, however, a study conducted in 2005/2006 reported that 20% of MSM engaged in unprotected, non-concordant anal sex in the previous 6 months^38^. A recent study on HIV transmission clusters in newly diagnosed PLWH from Croatia found that 86% (347 of 403) belonged to an HIV cluster of which the largest included 53 persons^39^. We could expect that similar clusters of syphilis have occurred during the current outbreak.

Risk factors associated with syphilis such as being MSM and having a history of previous syphilis have also been reported previously^22,23,27,40–43^. Younger age was also associated with syphilis; however, this relationship was not as strong as in other studies^40,43^. Interestingly, in the French Dat’AIDS cohort, age > 35 years was associated with a first or re-infection syphilis episode^28^ whereas, in the recent study from the US, syphilis incidence was highest in the 30–39 age group^28^. Of note, having a past history of clinical AIDS was related to a reduced risk for syphilis, this was also found in the univariable but not multivariable analysis in the French Dat’AIDS cohort^27^. There was no association of detectable HIV-1 viral load and syphilis which implies that the risky sexual behavior leading to syphilis will not increase in HIV cases. Some recent studies found an association of HCV infection with syphilis^23,28^; this was not the case in our study possibly because of a small number of HCV infections. Living in Zagreb the capital of Croatia was associated with syphilis mainly in our univariable analyses, syphilis outbreaks are especially common in large cities with significant populations of MSM^23,24,28,37,42^. The incidence of syphilis was higher in persons diagnosed with HIV in more recent years which might suggest that newer generations of MLWH have a different perception of risk and risky behavior. Similarly in a recent study from four US sites, a more recent entry into the cohort was associated with a higher incidence of syphilis^28^.

There was overall an increasing trend in the annual syphilis serologic testing rate from 2018 to 2021, however, the rate was lower in 2020 by 3.5% compared to 2019. Similarly, the proportion of men who had at least one annual syphilis test was lower in 2020 compared to 2019. It seems that measures to curb the COVID-19 epidemic had a negative impact on syphilis testing, similar suggestions on sexual health screening and patients’ referrals to STD clinics have also been made^44,45^. In Croatia, the first COVID-19 patient was documented on February 26, 2020, there have been several epidemic waves, and the highest number of confirmed cases was recorded in January and February 2021 (https://covid19.who.int/region/euro/country/hr, accessed Jun 7, 2023). A strict lockdown, including travel bans within the country, was implemented between March 19th and April 27th, 2020. This was followed by less strict measures (e.g. temporary closures of restaurants, bars, and fitness facilities, suspension of public events) that varied over time.

We have confirmed findings from others^46,47^ that repeated episodes of syphilis are more frequently asymptomatic. However, it is still debated whether this observation is a result of an immune response to a previous infection or an increased frequency of serological testing^47^. We found an increased likelihood of early latent syphilis in PLWH with a repeated syphilis episode in the background of a similar testing rate.

Our study has limitations. This was a retrospective longitudinal observational study, so causal inferences cannot reliably be made. We did not collect data on specific sexual practices, condom use, or other risky behaviors such as chemsex, methamphetamine use, group sex, transactional sex, or use of dating sites to find new partners. Hence, we cannot report on the risky behaviors that contributed to the rise in syphilis cases. The calculation of syphilis incidence depends on the definition of syphilis and testing. Our definition of a syphilis event was not solely based on laboratory confirmation of the infection with both a treponemal and non-treponemal test. Eighteen events did not have a positive RPR test, 12 of whom were in persons with signs of a genital ulcer and 6 were in asymptomatic persons who had TPHA seroconversion. All had an epidemiological link to a syphilis case and all received treatment for syphilis. Hence, by the inclusion of those cases, we might have slightly overestimated the incidence of syphilis. However, we also might have underestimated the incidence of syphilis by not including syphilis episodes at inclusion into care and by including in the study only participants who had at least a 1-year follow-up. We also included in the analysis persons with no annual syphilis tests because we did not want to exclude those at lower risk for syphilis infection. Finally, we might not have captured all syphilis tests done in our study population because persons might have done them in other healthcare facilities. However, it is unlikely that many persons in HIV care received a syphilis test outside UHID, because of the stigma surrounding HIV infection, MSM, and STDs in general in Croatia. The strength of the study is that we provided national data on syphilis incidence in men living with HIV in Croatia and provided convincing evidence of a huge rise in syphilis between 2019 and 2020. All our additional analyses including men who had annual syphilis tests and only MSM confirmed our main findings (Supplementary Tables 4S–6S and Supplementary Figs. 2–4).

## Conclusions

We report a 91% rise in the incidence of syphilis in men living with HIV in Croatia between 2019 and 2020. MSM were mainly affected. Traditional syphilis control strategies such as timely diagnosis and treatment, partner notification and treatment, and education/awareness campaigns might not be sufficient to curb the epidemic in men living with HIV We should strongly consider introducing doxycycline post-exposure prophylaxis which has been shown to significantly reduce syphilis in 3 randomized trials^48–50^. We should, however, also take into account that the wide use of doxycycline might increase antibacterial resistance and alter the microbiome, both of which are currently under investigation. Since many cases of syphilis are asymptomatic, serological screening should be increased particularly in those who had a previous syphilis episode. We showed that repeated episodes of syphilis are more frequently asymptomatic and not related to the frequency of testing. Longitudinal studies on the host humoral and cellular immune response in syphilis during index and repeated syphilis episodes could inform us about the underlying mechanisms of different clinical presentations.

## Supplementary Information

Supplementary Information.

## Data Availability

Data can be made available from the corresponding author upon reasonable request.
