# Supplementary material for: Incidence, risk factors, and clinical findings of syphilis among men living with HIV in Croatia during the COVID-19 pandemic
Source: Sci Rep. 2023 Jul 21;13:11784. doi: 10.1038/s41598-023-38807-1 (PMC10361976; doi:10.1038/s41598-023-38807-1)
Supplement: Supplementary file 1 — Supplementary Information. [file 41598_2023_38807_MOESM1_ESM.docx]

**­­­SUPPLEMENTARY MATERIAL**

**Age distribution:**

At the first-ever syphilis diagnosis on follow-up, the median age was lower (41.0 [Q1–Q3: 34.3–46.6, N=116 years]) than in MLWH with the recurrent syphilis event (median: 44.6 [Q1–Q3: 35.1–53.1 years, N=81]). Overall, the median age at first syphilis diagnosis at follow-up was 42.4 (Q1–Q3: 34.7–48.6, N=197) years. At the time of first syphilis diagnosis at follow-up, 67.0% (132 of 197) MLWH were 30 to 49 years old, 21.8% (43 of 179) were above 50 years and 11.2% (22 of 179) were younger than 30 years.

**Supplementary Table 1**. Frequency of syphilis testing in men living with HIV, Croatia, 2018 to 2021.

| Number of episodes of testing per calendar year | Calendar year | | | |
| --- | --- | --- | --- | --- |
|  | 2018 | 2019 | 2020 | 2021 |
| None | 218 (22.2) | 136 (12.1) | 230 (19.6) | 172 (15.1) |
| 1 | 687 (69.8) | 810 (71.7) | 707 (60.4) | 466 (40.8) |
| 2 | 60 (6.1) | 152 (13.5) | 190 (16.2) | 425 (37.3) |
| ≥ 3 | 19 (1.9) | 31 (2.8) | 44 (3.8) | 78 (6.8) |

Values are frequencies and percentages (in parenthesis). An episode of syphilis testing includes a *Treponema pallidum* hemagglutination test with or without a Rapid Plasma Regain test. Men were males at birth, there were 3 transgender women.

**Supplementary Table 2**. Frequency of syphilis testing in men having sex with men living with HIV, Croatia, 2018 to 2021.

| Number of episodes of testing per calendar year | Calendar year | | | |
| --- | --- | --- | --- | --- |
|  | 2018 | 2019 | 2020 | 2021 |
| None | 157 (19.6) | 91 (9.9) | 152 (15.7) | 118 (12.5) |
| 1 | 571 (71.2) | 660 (71.7) | 593 (61.3) | 365 (38.5) |
| 2 | 55 (6.9) | 138 (15.0) | 179 (18.5) | 389 (41.1) |
| ≥ 3 | 19 (2.4) | 31 (3.4) | 44 (4.5) | 75 (7.9) |

Values are frequencies and percentages (in parenthesis). An episode of syphilis testing includes a *Treponema pallidum* hemagglutination test with or without a Rapid Plasma Regain test. Men were males at birth, there were 3 transgender women.

**Supplementary Table 3.** Comparison of men living with HIV with an annual syphilis test in all calendar years under follow-up *versus* those

who had at least one calendar year with no syphilis tests performed, Croatia 2018 to 2021.

|  | Had a calendar year without a syphilis test | |  | |
| --- | --- | --- | --- | --- |
|  | No (N = 674) | Yes (N = 513) | Total (N = 1187) | P Value |
| Age, years | 40.1 (32.8 – 47.7) | 43.9 (37.2 – 53.0) | 41.7 (34.6–50.4) | <0.001 |
| Mode of transmission |  |  |  | <0.001 |
| MSM | 603 (89.5) | 373 (72.7) | 0976 (82.2) |  |
| Heterosexual | 050 (7.4%) | 092 (17.9) | 0142 (12.0) |  |
| Other/unknown | 021 (3.1) | 048 (9.4) | 0069 (5.8) |  |
| Known duration of HIV infection, years | 3.0 (0.2 – 7.7) | 7.1 (2.7 – 12.9) | 4.6 (1.1–10.3) | <0.001 |
| Living in Zagreb |  |  |  | <0.001 |
| No | 310 (46.0) | 308 (60.0) | 0618 (52.1) |  |
| Yes | 364 (54.0) | 205 (40.0) | 0569 (47.9) |  |
| Duration of antiretroviral therapy | 2.2 (0.0 – 6.3) | 5.4 (2.1 – 10.8) | 3.5 (0.8 – 8.4) | <0.001 |
| Had syphilis at or before inclusion^a^ |  |  |  | 0.100 |
| No | 621 (92.1) | 485 (94.5) | 1106 (93.2) |  |
| Yes | 053 (7.9) | 028 (5.5) | 0081 (6.8) |  |

Values are frequencies and percentages (in parenthesis). MSM, men who have sex with men. Men were males at birth, there were 3 transgender women.

^a^Based on history and/or positive *Treponema pallidum* hemagglutination assay.

**Supplementary Table 4.** Rates of new symptomatic and asymptomatic syphilis events among 1159 men living with HIV who had at least one syphilis test done

annually, Croatia, 2018 to 2021.

| Year | Symptomatic syphilis | | Asymptomatic syphilis | | Total syphilis events | |
| --- | --- | --- | --- | --- | --- | --- |
|  | Rate per 100 PY (95 % CI) | Percent change from preceding year | Rate per 100 PY (95 % CI) | Percent change from preceding year | Rate of syphilis  per 100 PY (95 % CI) | Percent change from preceding year |
| 2018 | 2.7 (1.7–4.2) | – | 1.4 (0.8–2.6) | – | 4.1 (2.8–5.9) | – |
| 2019 | 2.6 (1.7–3.8) | -1.5 | 1.7 (1.0–2.8) | 18.2 | 4.3 (3.1–5.8) | 5.5 |
| 2020 | 5.6 (4.2–7.3) | 118.5 | 3.2 (2.2–4.6) | 86.2 | 8.8 (7.0–11.0) | 105.1 |
| 2021 | 6.2 (4.8–8.0) | 12.0 | 4.6 (3.4–6.2) | 46.5 | 10.8 (8.9–13.1) | 25.2 |

Men were males at birth, there were 3 transgender women. Percent change from preceding year based on the incidence rate ratio from

generalized estimating equations models. PY, person-years. CI, confidence interval.

**Supplementary Table 5.** Rates of new symptomatic and asymptomatic syphilis events among 976 men having sex with men living with HIV,

Croatia, 2018 to 2021.

| Year | Symptomatic syphilis | | Asymptomatic syphilis | | Total syphilis events | |
| --- | --- | --- | --- | --- | --- | --- |
|  | Rate per 100 PY (95 % CI) | Percent change from preceding year | Rate per 100 PY (95 % CI) | Percent change from preceding year | Rate of syphilis  per 100 PY (95 % CI) | Percent change from preceding year |
| 2018 | 2.5 (1.6–3.9) | – | 1.5 (0.8–2.6) | – | 4.0 (2.8–5.7) | – |
| 2019 | 2.6 (1.8–4.0) | 4.8 | 1.7 (1.0–2.9) | 15.1 | 4.4 (3.2–6.0) | 8.3 |
| 2020 | 5.5 (4.2–7.2) | 113.3 | 2.9 (2.0–4.2) | 66.7 | 8.4 (6.7–10.5) | 94.4 |
| 2021 | 6.2 (4.8–8.0) | 12.4 | 4.3 (3.2–5.9) | 50.5 | 10.5 (8.6–12.8) | 25.9 |

Men were males at birth, there were 3 transgender women. Percent change from preceding year based on the incidence rate ratio from

generalized estimating equations models. PY, person-years. CI, confidence interval.

**Supplementary Table 6.** Rates of new symptomatic and asymptomatic syphilis events among 962 men having sex with men living with HIV who had

at least one syphilis test done annually, Croatia, 2018 to 2021.

| Year | Symptomatic syphilis | | Asymptomatic syphilis | | Total syphilis events | |
| --- | --- | --- | --- | --- | --- | --- |
|  | Rate per 100 PY (95 % CI) | Percent change from preceding year | Rate per 100 PY (95 % CI) | Percent change from preceding year | Rate of syphilis  per 100 PY (95 % CI) | Percent change from preceding year |
| 2018 | 3.0 (1.9–4.8) | – | 1.7 (0.9–3.1) | – | 4.7 (3.2–6.8) | – |
| 2019 | 2.9 (2.0–4.4) | -1.0 | 1.9 (1.2–3.2) | 10.9 | 4.9 (3.5–6.7) | 3.2 |
| 2020 | 6.4 (4.9–8.5) | 124.2 | 3.5 (2.4–5.1) | 79.3 | 9.9 (7.9–12.4) | 106.0 |
| 2021 | 7.0 (5.4–9.1) | 9.0 | 4.9 (3.6–6.7) | 43.8 | 11.9 (9.8–14.6) | 21.4 |

Men were males at birth, there were 3 transgender women. Percent change from preceding year based on the incidence rate ratio from

generalized estimating equations models. PY, person-years. CI, confidence interval.

**
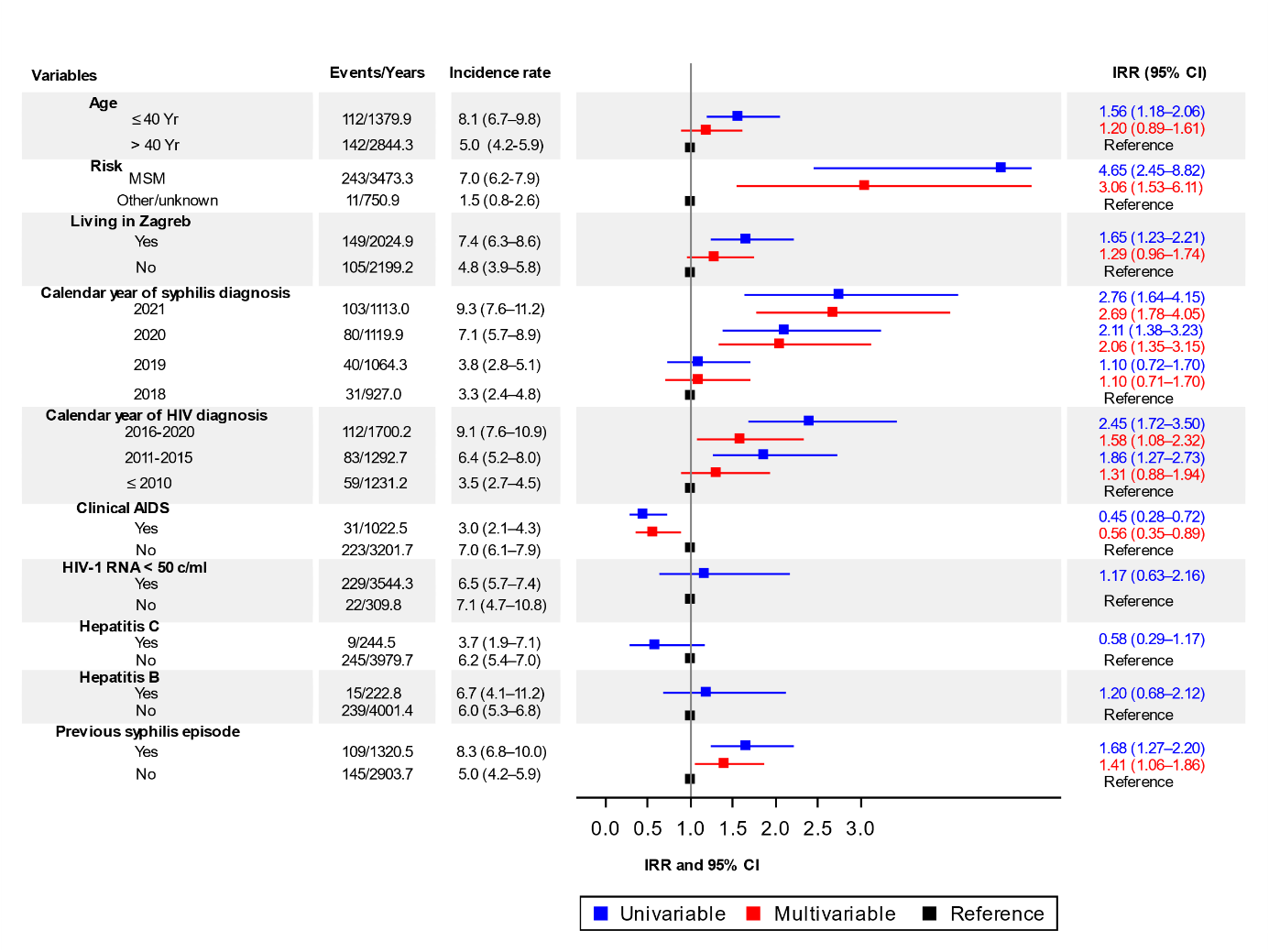
**

**Supplementary Figure 1:** Rates of incident syphilis per 100 PYFU with 95% confidence interval in 1187 men living with HIV by sociodemographic and clinical characteristics and crude (univariable) and multivariable IRR for syphilis events, Croatia, 2018 to 2021. Years are person-years of follow-up. The overall incidence rate per 100 PYFU was 6.0 (95% CI, 5.3–6.8).

Men were males at birth, there were 3 transgender women. MSM, men who have sex with men; IRR, incidence rate ratio; CI, confidence intervals PYFU, person-years of follow-up; Clinical AIDS, HIV-1 RNA, hepatitis C, and previous syphilis diagnosis were time-updated variables. IRR are based on a generalized estimating equations model with an exchangeable correlation structure. The upper 95% CI for risk is truncated.

The 95% confidence intervals for the rate per 100 person-years of follow-up was calculated using the Rothman/Greenland estimation.

**
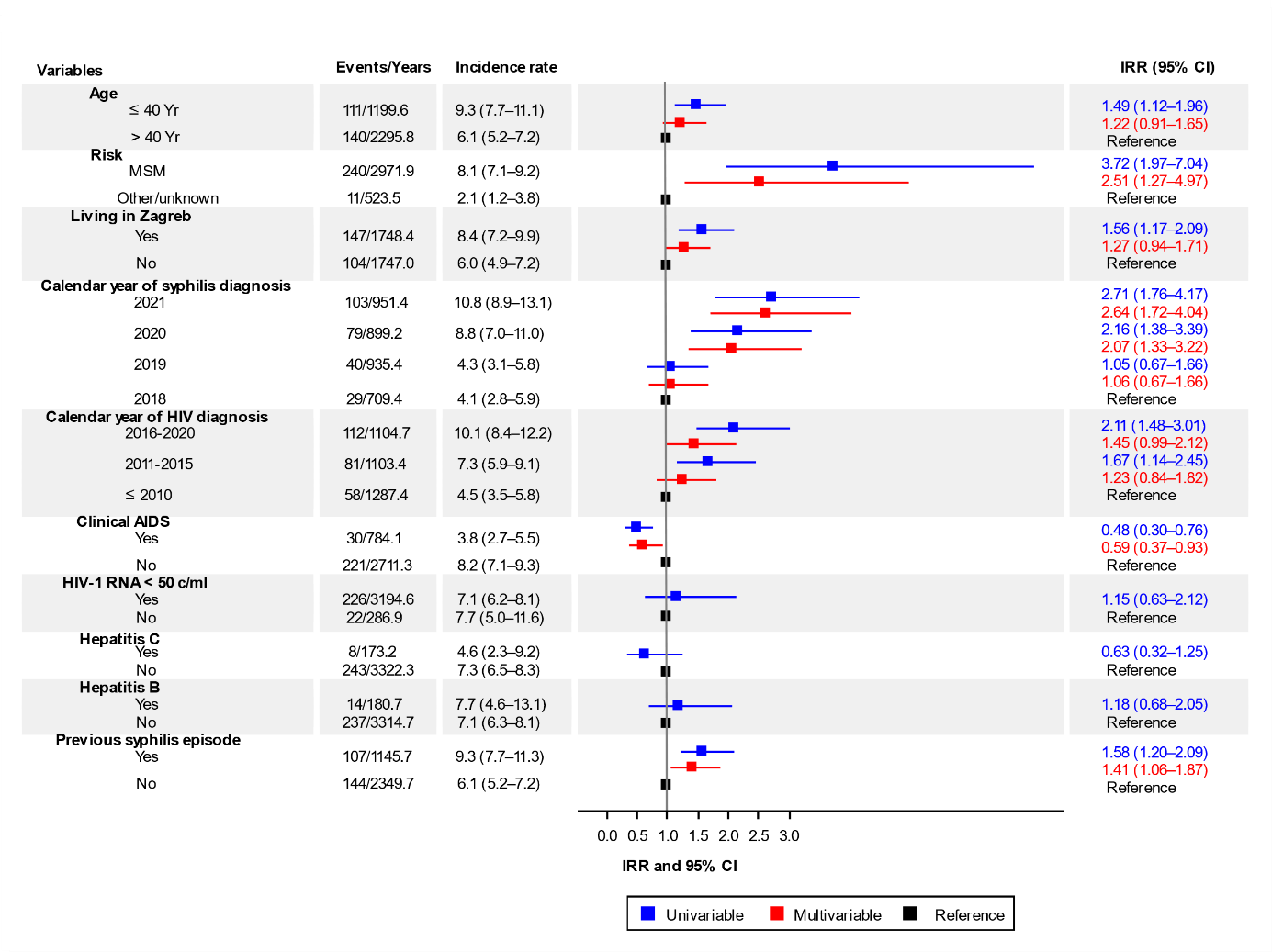
**

**Supplementary Figure 2:**

Rates of incident syphilis per 100 PYFU with 95% confidence interval in 1159 men living with HIV who had at least one syphilis test done annually by sociodemographic and clinical characteristics and crude (univariable) and multivariable IRR for syphilis events, Croatia, 2018 to 2021. Years are person-years of follow-up. The overall incidence rate per 100 PYFU was 7.2 (95% CI, 6.3–8.1).

Men were males at birth, there were 3 transgender women. MSM, men who have sex with men; IRR, incidence rate ratio; CI, confidence intervals; PYFU, person-years of follow-up; Clinical AIDS, HIV-1 RNA, hepatitis C, and previous syphilis diagnosis were time-updated variables. IRR are based on a generalized estimating equations model with an exchangeable correlation structure. Calendar years with no syphilis tests were excluded. The 95% confidence intervals for the rate per 100 person-years of follow-up was calculated using the Rothman/Greenland estimation.

**
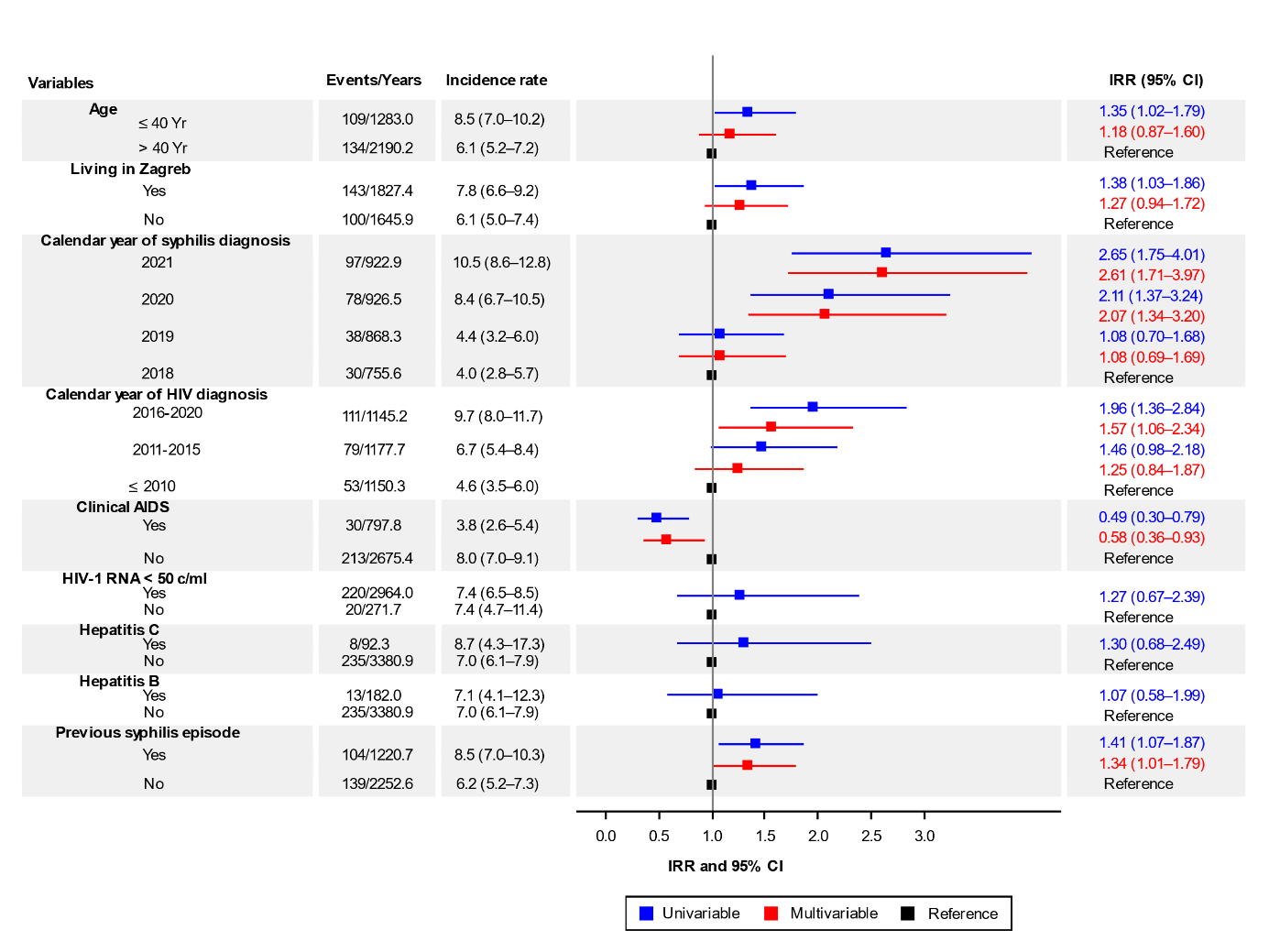
**

**Supplementary Figure 3:**

Rates of incident syphilis per 100 PYFU with 95% confidence interval in 976 MSM living with HIV by sociodemographic and clinical characteristics and crude (univariable) and multivariable IRR for syphilis events, Croatia, 2018 to 2021. Years are person-years of follow-up. The overall incidence rate per 100 PYFU was 7.0 (95% CI, 6.2–8.0).

Men were males at birth, there were 3 transgender women. MSM, men who have sex with men; IRR, incidence rate ratio; CI, confidence intervals; PYFU, person-years of follow-up; Clinical AIDS, HIV-1 RNA, hepatitis C, and previous syphilis diagnosis were time-updated variables. IRR are based on a generalized estimating equations model with an exchangeable correlation structure. The 95% confidence intervals for the rate per 100 person-years of follow-up was calculated using the Rothman/Greenland estimation.

**
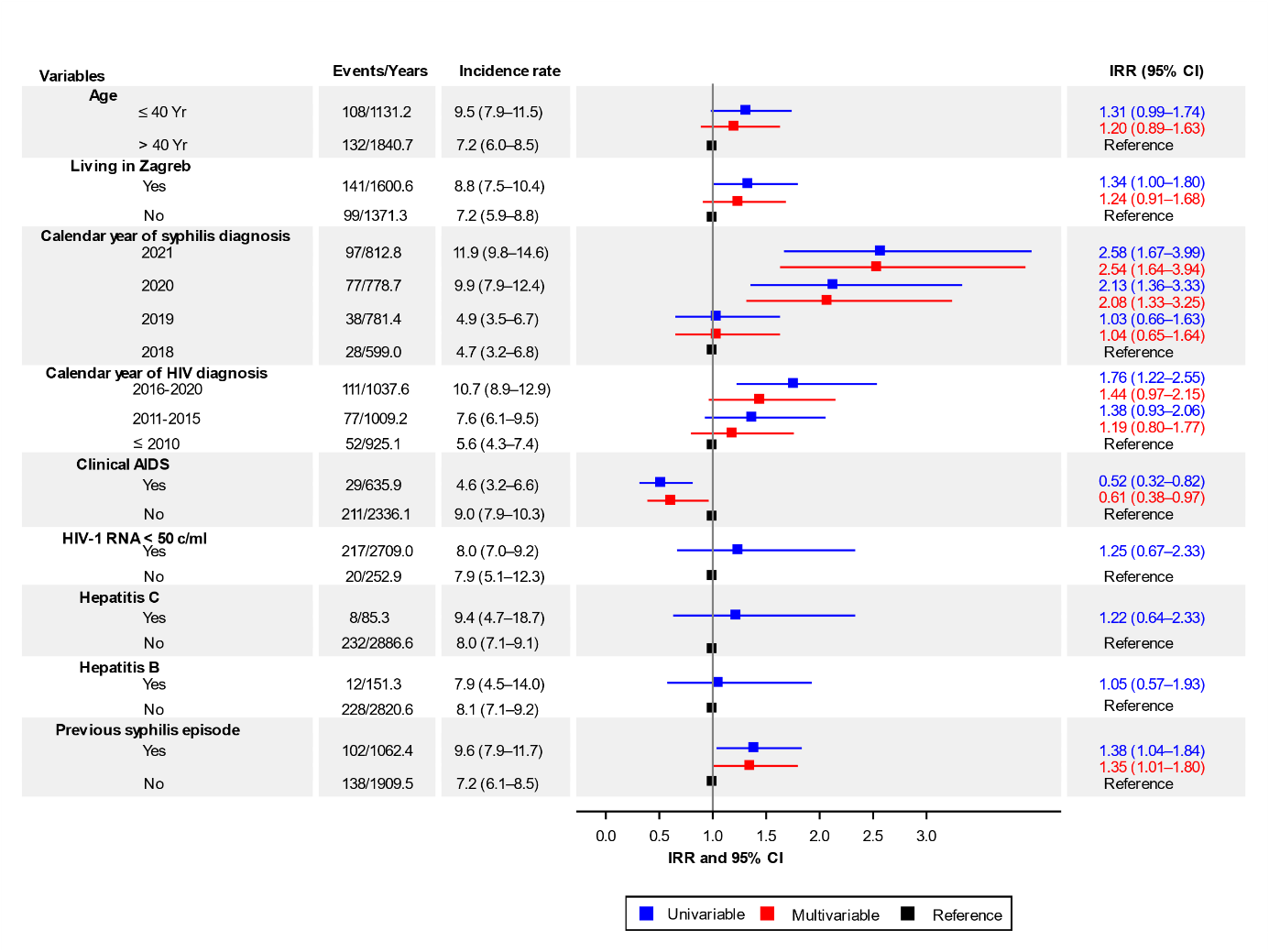
**

**Supplementary Figure 4:** Rates of incident syphilis per 100 PYFU with 95% confidence intervals in 962 MSM living with HIV who had at least one syphilis test done annually by sociodemographic and clinical characteristics and crude (univariable) and multivariable IRR of syphilis events, Croatia, 2018 to 2021. Years are person-years of follow-up. The overall incidence rate per 100 PYFU was 8.1 (95% CI, 7.1–9.2).

Men were males at birth, there were 3 transgender women. MSM, men who have sex with men; IRR, incidence rate ratio; CI, confidence intervals; PYFU, person-years of follow-up; Clinical AIDS, HIV-1 RNA, hepatitis C, and previous syphilis diagnosis were time-updated variables. IRR are based on a generalized estimating equations model with an exchangeable correlation structure. Calendar years with no syphilis tests were excluded. The 95% confidence intervals for the rate per 100 person-years of follow-up was calculated using the Rothman/Greenland estimation.
